# Minimally invasive plate osteosynthesis for complex comminuted bone fractures in the Fraser’s type II floating knee: a case report

**DOI:** 10.1186/s40001-022-00649-4

**Published:** 2022-03-02

**Authors:** Chuangang Peng, Guangkai Ren, Minghan Dou, Baoming Yuan, Dankai Wu

**Affiliations:** Orthopaedic Medical Center, The Second Hospital of Jilin University, No 218, Lane Ziqiang, Nanguan District, Changchun, 130041 People’s Republic of China

**Keywords:** Case report, Complex comminuted bone fractures, Femoral mid-distal segments, Floating knee

## Abstract

**Objective:**

Floating knee type IIC, according to Fraser’s classification, is an uncommon severe injury that typically occurs in polytrauma. In such cases, intra-articular fracture and the high degree of comminution and deformity of the mid-distal femur make fixation challenging. The purpose of this study was to demonstrate that minimally invasive plate osteosynthesis (MIPO) technology can simplify these complex problems and improve patient prognosis.

**Case presentation:**

A 38-year-old man injured his left leg in a car accident, causing pain, swelling, deformity, and limited mobility on his left knee and thigh, and two small open wounds were noted mainly of the anterior aspect of the mid-distal thigh. Physical examination and computed tomography angiography of the lower limb confirmed that there was no damage to the neurovascular system. The clinical diagnosis was closed intra-articular fracture of the proximal tibia, open intra-articular fracture of the distal femur with extension to the diaphysis, and a patellar fracture on the ipsilateral knee. The treatment strategy involved a locking plate system applying MIPO technology. Postoperative evaluation of the patient was satisfactory, with immediate functional exercise, full weight-bearing after three months, and return to daily activity without pain. Final follow-up taken 3 years after surgery showed good lower limb alignment and complete plasticity of the bone structure, by which time the patient showed good limb function.

**Conclusions:**

Minimally invasive techniques can provide a simple and effective treatment for some complex fractures.

## Introduction

Ipsilateral fractures of the femur and tibia, also known as floating knee, are uncommon severe injuries that typically occur in polytrauma. In some series, poor results have been reported, particularly when both fractures are intra-articular (floating knee type IIC, according to Fraser’s classification) [1]. In such cases, operative treatment is mandatory to restore articular congruency and overall leg alignment. Recent reports have shown that surgical stabilization of both fractures and early mobilization can avoid most complications and achieve optimal clinical results [2–4].

The purpose of this study was to evaluate the operative results and functional outcomes following surgical treatment of a case of severe floating knee. The main features of the case presented here were complex comminuted fractures and malformations of the mid-distal femur, which make local fixation and treatment very difficult.

## Case presentation

### Ethical approval

The study was conducted in accordance with the principles outlined in the Declaration of Helsinki and was approved by the Ethics Committee of the Second Hospital of Jilin University. The patient provided written informed consent for participation. Data anonymity was maintained to protect patient privacy.

### Patient characteristics

A 38-year-old man injured his left leg in a car accident, sustaining a closed intra-articular fracture of the proximal tibia and open intra-articular fracture of the distal femur with extension to the diaphysis. A patellar fracture was observed in the ipsilateral knee. Two small open wounds were noted mainly on the anterior aspect of the mid-distal femur. The level of contamination was minimal, and there were no soft-tissue defects that needed to be covered with a flap. Initial management consisted of debridement and application of bone traction. The soft tissue settled after a week. Physical examination and lower limb computed tomography angiography confirmed normal neurovascular status. The patient reported no history of illness or surgery, and no family members had any inherited diseases.

**Fig. 1:**
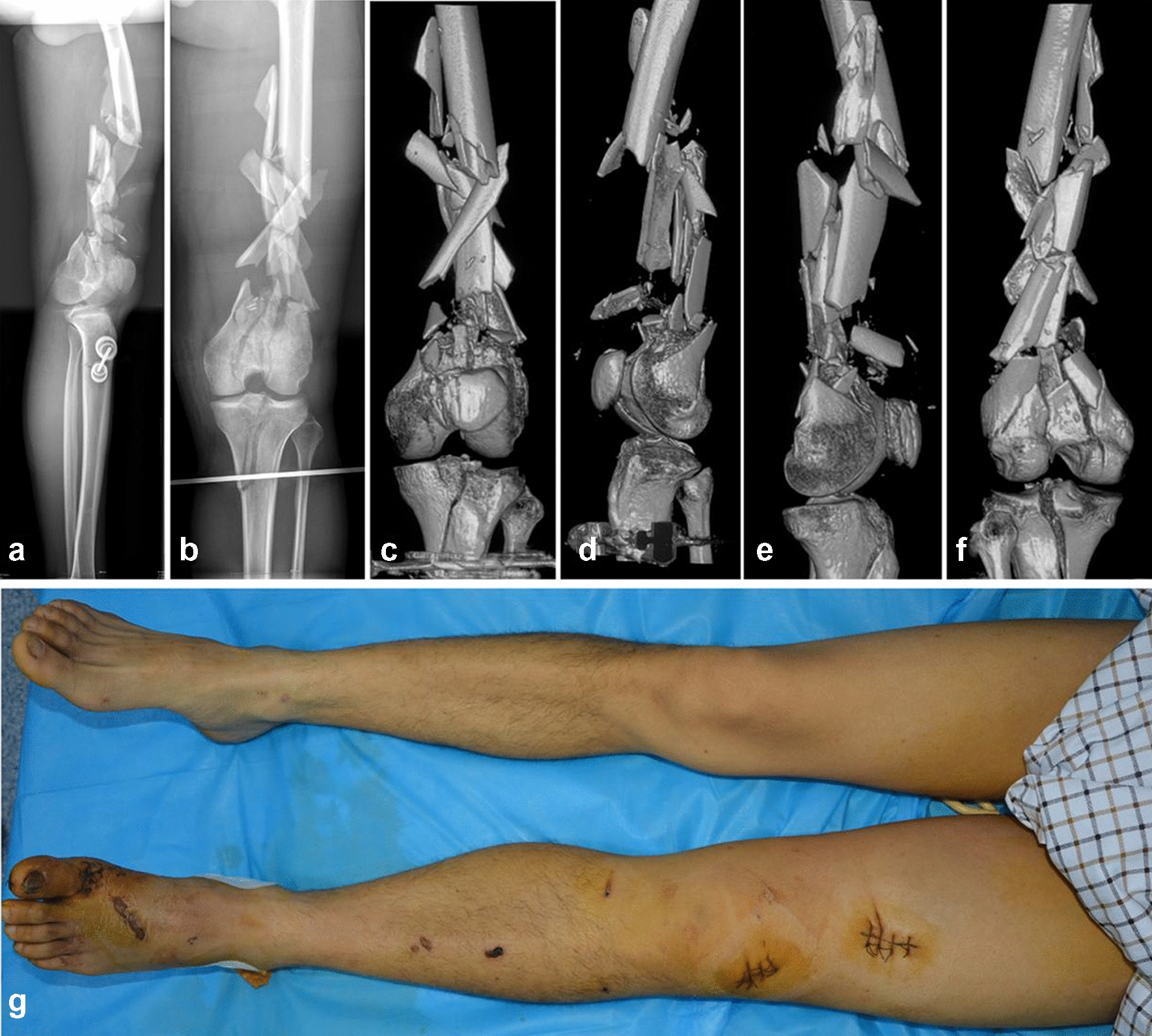
Preoperative images. **a**, **b** Preoperative radiographs: anterior view and lateral view of the lower limb. **c**–**f** Preoperative three-dimensional CT scans. **g** Preoperative soft-tissue condition around the knee joint

Preoperative radiographs showed multifragmentary fracture of the distal femur, involving both condyles and extending into the diaphysis. A sagittal split of the medial condyle was observed. Three-dimensional computed tomography (3D CT) scans showed the extent and location of the articular fractures (Fig. 1). Furthermore, the medial condyle fracture was separated and displaced. The reparation of large segmental comminuted bone fractures of the femoral mid-distal segments are the most complex part of the operation. Supracondylar and shaft fracture of the left femur exposed Gustilo Anderson II, with minor (< 5%) bone loss of the metaphyseal area.

The distal femoral fracture was classified as type 31C3.3, and the patellar fracture was classified as type 34B2.2 according to the AO Foundation and the Orthopedic Trauma Association system; the proximal tibia fracture was classified as Schatzker type IV.

**Fig. 2:**
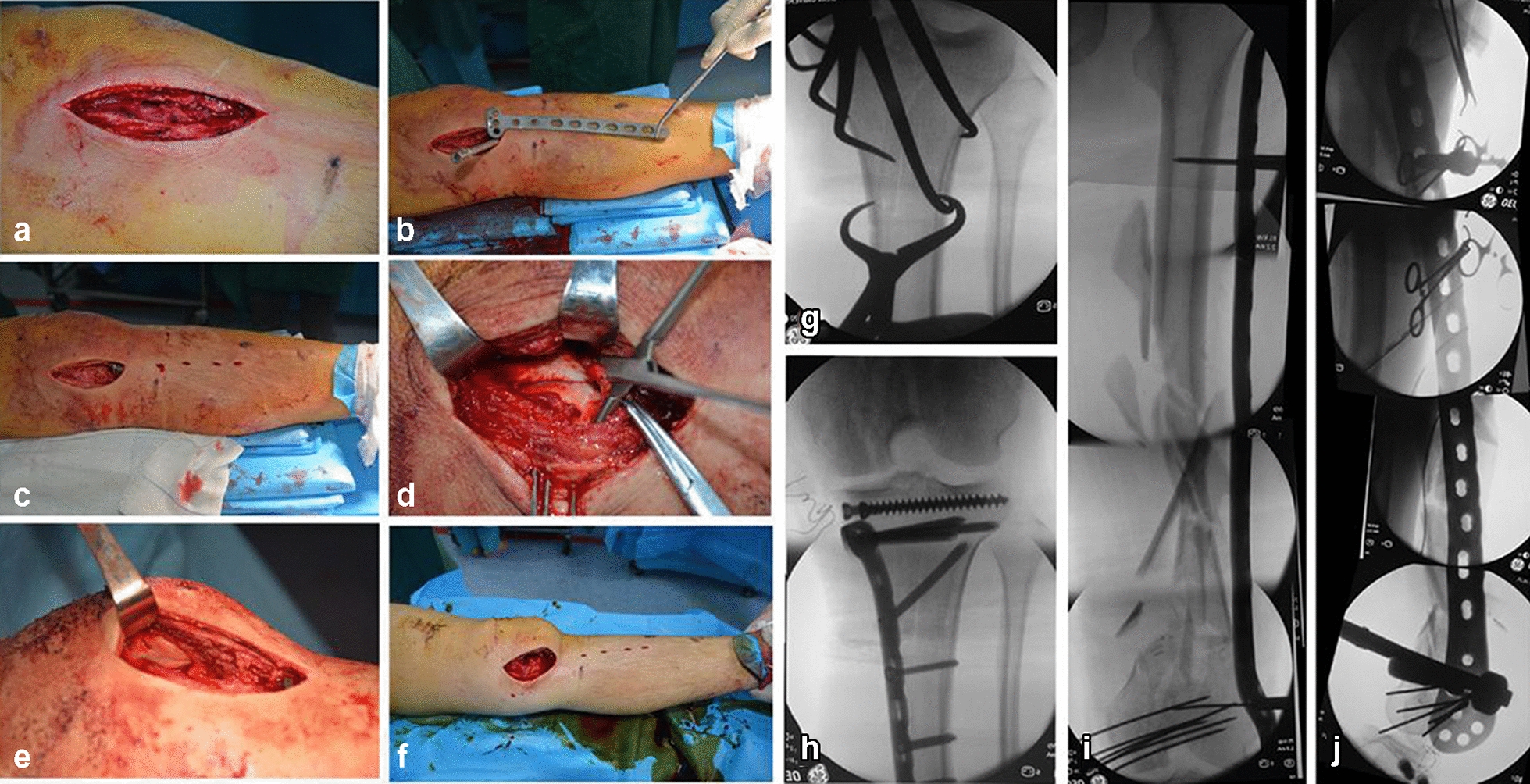
Surgical procedures. **a** Medial parapatellar approach. **b**, **c** Percutaneous placement of the proximal medial tibia plate. **d** Reduction and temporary fixation of the femoral condyle fracture under direct vision. **e** Exposing and fixing the patellar fracture. **f** A modified standard lateral approach. **g**–**h** Intraoperative imaging showing reduction and fixation of tibial plateau fractures. **i**, **j** Intraoperative fluoroscopy of the femoral fracture force line and plate position

The patient underwent surgery to restore articular congruency and the overall alignment of leg. Debridement of the wound and skeletal traction were planned as initial management, because of the open fracture. Intramedullary nailing was difficult because of the fracture morphology. The MIPO technique was chosen for treatment of the complex floating knee injuries (Fig. 2).

After induction of general anesthesia, the patient was positioned supine on a radiolucent table, with both legs draped free and a support placed under the injured knee. A medial parapatellar approach was adopted, and decisions made based on the location of the sagittal fracture separation of the femoral medial articular surface. A medial parapatellar approach was also used to fix the associated patellar fracture (34 B2.2); after repositioning of the patella, two 2.0-mm cortical bone screws were used to fix the patella fracture. Tibial fractures (Schatzker IV) were treated using a medial patella approach combined with the MIPO technique. Indirect percutaneous articular reduction was performed using pointed reduction forceps and a subchondral raft of 4.5-mm cancellous screws. A locking medial plate was slipped through the medial parapatellar approach, and percutaneous locking screws inserted to complete the fixation.

The focus of this article is on treatment of the femoral mid-distal segments using a distal femoral locking compression plate (LCP-DF) and the MIPO technique. Slight eversion of the patella and flexion of the knee through the medial parapatellar approach exposed the distal articular surface, particularly of the medial femoral condyle, thereby enabling reduction and fixation of the articular fractures. Large pointed reduction forceps were useful to hold the medial and lateral femoral condyles together after reduction. A Kirschner wire was inserted into the medial condyle for temporary fixation of the articular surface fractures. The distal femoral fracture changed from C3 to A3 type.

A modified standard lateral approach was used for extra-articular fractures. The implant was slipped into a submuscular tunnel along the lateral cortex of the femur. A minimum 7-hole length coverage over the shaft fixation with five screws is optimum. No screw could be placed in the middle segment; therefore, in this case, a 14-hole proximal tibia locking compression plate was chosen. The distal part of the LCP-DF was aligned according to the anatomical reference with the distal femur, and the proximal part of the plate then ran along the femoral shaft. To obtain a closed reduction, traction was performed after temporary fixation of the femoral condyle with the distal part of the plate. Manual traction was applied to the ankle with a force vector directed posteriorly using the supracondylar pad as a fulcrum to help reduce the fracture and restore limb length and rotational and axial alignments. Fluoroscopic images were used to recheck the position. Final fixation was achieved using locking screws at either end of the plate. A total of seven 5.0 mm locking screws were placed into the distal fragment and five screws were placed in the shaft fragment. This construct aimed to combine absolute stability with fixed angle stability and relative stability using bridging technology. After fixation, the stability of the knee joint should always be checked. Limb length, axes, and rotation were checked using clinical and radiological methods.

Bone defects were observed in the middle fragment. Bone grafting to correct the fragment loss was not performed, because the loss did not affect the length or alignment of the femur, and the entire construct was stable, with balanced fixation, as described above.

A final check of fracture reduction and fixation was performed using an image intensifier. Overall, the construct achieved balanced and adequate fixation. After internal fixation, passive exercises were performed on the patient under anesthesia.

## Outcomes and follow-up

**Fig. 3:**
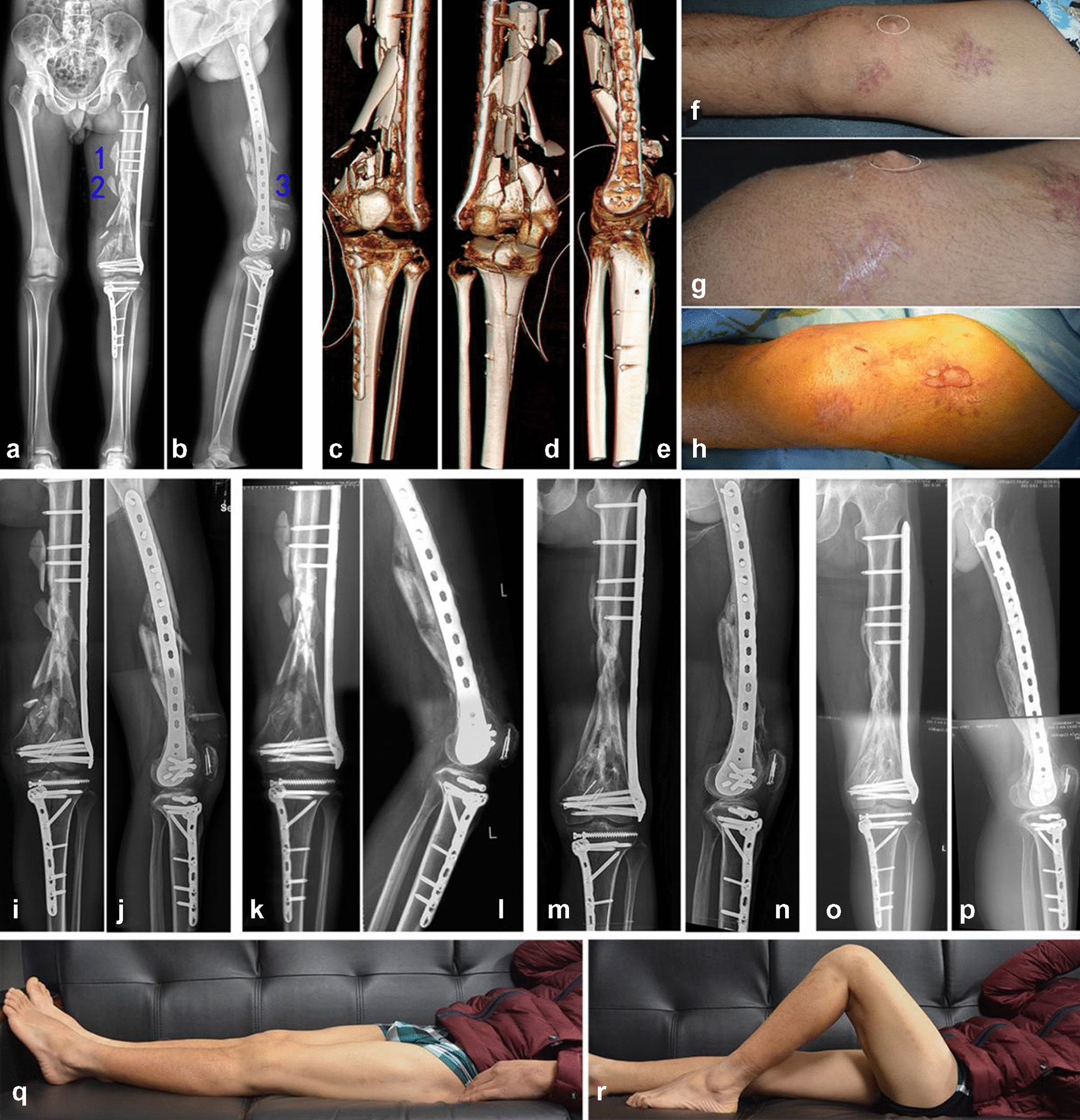
Postoperative images. **a**–**e** Immediate postoperative radiographs and three-dimensional CT scans demonstrate correct mechanical axis and good plate position. **f**, **g** Unreduced reverse fragment of the femoral metaphysis. **h** A bone fragment was removed through a small incision at 1.5 months postoperatively. **i**–**p** Radiographic examinations at follow-up. 1.5 months (**i**, **j**), 3 months (**k**, **i**), 1 years (**m**, **n**), and 3 years (**o**–**p**) post-operation. **q**–**r** Clinical images showing functional outcomes at the last follow-up

Postoperative 3D CT and radiography confirmed that the intra-articular fractures underwent anatomic reduction, and the left femur line was restored, with internal fixation in a good position; however, there were three unreduced reverse fragments (1, 2, and 3) in the femoral mid-distal segment after plating **(**Fig. 3a–e). On the first postoperative day, the patient began continuous passive range-of-motion exercises under the guidance of an orthopedic surgeon. The patient was allowed to walk on crutches with toe-touch weight-bearing during the first 6 weeks. Because of the need for functional exercise, the unreduced reverse fragment (3) was removed 1.5 months postoperatively through small incisions. Based on fracture healing assessed on radiographs at 1.5 months, weight-bearing was progressively increased to full weight-bearing at 3 months. Radiographic examinations for 3 months showed no displacement at the fracture end, and the fracture line was blurred by the formation of a large amount of callus. Further radiographic examinations after 1 year showed good lower limb alignment and good plasticity of the bone structure. Final follow-up at 3 years showed good lower limb alignment and complete plasticity of the bone structure, by which time the patient showed good limb function (acceptable range of knee motion of 0°–100° was achieved). Although fractures of the middle and lower femur were healed, the bone strength was insufficient; hence, implant removal will not be considered (Fig. 3i–r).

## Discussion and conclusions

“Floating knee” injury is often the result of high-energy trauma, which may lead to complex fractures and extensive soft-tissue injuries [4]. In this case, we encountered ipsilateral fractures of the tibial plateau and femoral condyle featuring extensive articular involvement, which are very rare in floating knee type IIc injuries (Fraser’s classification). In particular, extremely comminuted fracture morphology of the mid-distal femur is clinically rare. Reconstruction of complex comminuted bone fractures of femoral mid-distal segments by open reduction is technically challenging and can lead to a significant stripping of soft tissue. Successful surgery depends on effective reduction and internal fixation of the fracture, with maximal preservation of the blood supply at the fracture site [5].

Minimally invasive osteosynthesis is a commonly performed technique designed to provide stable internal fixation through a minimally invasive surgical technique. Intramedullary nailing is difficult occasionally because the fracture morphology involves the articular surface in this fracture pattern. MIPO is the preferred approach in clinical situations where the fracture involves the articular surface.

For complex comminuted bone fractures, flexible fixation using the bridge plate concept without touching the fracture zone is preferred. The length of the fracture comminution usually determines the bridging length or working length of the plate. A plate with insufficient supporting length is considered main cause of fracture nonunion and internal fixation fractures [6]. Again, as the patient in this report had comminuted distal femoral fractures with diaphyseal involvement comprising a large segmental component, plates of appropriate length were chosen to achieve sound mechanical fixation, which is key to facilitating healing through callus formation. Hence, according to these principles, we chose a 14-hole plate for our patient, to achieve a ‘balanced fixation construct’. One anatomical study showed some discrepancies between the side plate and the femoral shaft [7]; given this potential mismatch, surgeons may have to consciously accept a certain gap between the plate and the bone when using a 9-hole or longer LCP-DF. In our case, there was a clear gap between the 14-hole plate and the femoral diaphyseal fracture. The LCP was fixed to the bone and a space of 0.5–1 cm between the bone and the plate left, to preserve the periosteum. In cases with relative stability multifragmentary fractures, the reserved gap allows micromotion to induce callus formation.

A decision on whether an additional medial plate should be added must be made during the operation. Double plating is proven to be stronger than a single lateral plate in biomechanical testing; however, it is not superior to traditional lateral plating with respect to clinical outcomes [8–10]. In our patient, we decided not to add a medial plate for the following reasons: the single lateral locking plate was strong enough to maintain sufficient stability to allow functionality after treatment; further, the double-plate technique is associated with a risk of delayed union or nonunion due to extensive soft-tissue dissection and periosteal injury, even when performed by skilled surgeons; finally, when the overall structure is balanced and stable, lateral-only locked plates can reduce surgical time, blood loss, and limit soft-tissue stripping.

Restoration of limb alignment is critical to produce an optimal outcome for stabilization of complex floating knee injuries. Malalignment of the femur and tibia in any plane can lead to abnormal load transmission across the adjacent joints, resulting in shearing of the articular cartilage and development of earlier joint arthrosis, leading to clinically relevant degenerative changes [11, 12]. Nevertheless, with respect to the metaphyseal/diaphyseal component, clinicians should be more willing to accept basic restoration of the femoral line, rather than anatomic reduction of all fracture fragments. A minor residual gap after closed reduction does not appear to result in a higher risk of nonunion; however, in the open technique, anatomical reduction of numerous small pieces is extremely challenging and risks devitalizing the free bone fragments [4, 13]. In our patient, there were three free fragments that presented a reversed morphology, in that the endosteum was turned inside out. It is possible that neo-vascularization to the callus was compromised as the periosteal callus was set in the opposite direction given the reversed morphology [14]. One of the unreduced reverse fragments was removed 1.5 months after surgery, and the remaining two fragments began to show signs of necrotic absorption three months after surgery, until they were completely absorbed. Finally, an acceptable healing strength was achieved for the entire lower limb.

The advantages of indirect fracture reduction and submuscular plating in the treatment of floating knee injury lie in the fact that the soft tissue envelope surrounding the fracture remains largely intact, thus preserving the biology, enhancing the chances of fracture union and reducing the need for bone grafting and the incidence of infection. The surgical technique described in this report had some limitations. First, the style of bone graft requiring adequate exposure of the graft site in the metadiaphyseal component is limited by the minimally invasive incision used in MIPO technology. Second, minimally invasive techniques with indirect fracture reduction can lead to higher rates of limb malalignment, and improved reduction methods to avoid deformity, especially malrotation, and repeated intraoperative fluoroscopy, to check the restoration should be advocated. Third, the anterior unreduced reverse fragment affected knee flexion movement in the early postoperative period, because its tip was inserted into the quadriceps muscle (Fig. 3f–h). Finally, the healing strength of a femoral fracture is partially affected by the reduced bone mass; thus, implant removal is prone to refracture.

The advantages of MIPO for treating complex floating knee injuries are rapid and optimal healing, minimization of soft-tissue complications and loss of function, and prevention of nonunion or malunion. In particular, the recommended current method of a single lateral locking plate using MIPO technology ensures good outcomes for comminuted bone fractures of the femoral mid-distal segments.

## Data Availability

The datasets used and analyzed during the current study are available from the corresponding author on reasonable request.
